# Genetic and Epigenetic Impact of Chronic Inflammation on Colon Mucosa Cells

**DOI:** 10.3389/fgene.2021.722835

**Published:** 2021-10-26

**Authors:** Jia He, Jimin Han, Jia Liu, Ronghua Yang, Jingru Wang, Xusheng Wang, Xiaodong Chen

**Affiliations:** ^1^ Department of Burn Surgery, The First People’s Hospital of Foshan, Foshan, China; ^2^ School of Pharmaceutical Sciences (Shenzhen), Sun Yat-sen University, Guangzhou, China

**Keywords:** chronic colitis, chronic inflammation, SNP/indel, DNA methylation, cancer, aging

## Abstract

Chronic inflammation increases cancer risk, and cancer development is characterized by stepwise accumulation of genetic and epigenetic alterations. During chronic inflammation, infectious agents and intrinsic mediators of inflammatory responses can induce genetic and epigenetic changes. This study tried to evaluate both the genetic and epigenetic influence of chronic inflammation on colon mucosa cells. Repetitive dextran sulfate sodium (DSS) treatment induced chronic colitis model. Whole-exome sequencing (WES) (200× coverage) was performed to detect somatic variations in colon mucosa cells. With the use of whole-genome bisulfite sequencing (BS) at 34-fold coverage (17-fold per strand), the methylome of both the colitis and control tissue was comparatively analyzed. Bioinformatics assay showed that there was no significant single-nucleotide polymorphism/insertion or deletion (SNP/InDel) mutation accumulation in colitis tissue, while it accumulated in aged mice. Forty-eight genes with SNP/InDel mutation were overlapped in the three colitis tissues, two (Wnt3a and Lama2) of which are in the cancer development-related signaling pathway. Differentially methylated region (DMR) assay showed that many genes in the colitis tissue are enriched in the cancer development-related signaling pathway, such as PI3K–AKT, Ras, Wnt, TGF-beta, and MAPK signaling pathway. Together, these data suggested that even though chronic inflammation did not obviously increase genetic mutation accumulation, it could both genetically and epigenetically alter some genes related to cancer development.

## Introduction

Chronic inflammation has been indicated as an important risk factor for cancer; one of the best examples of the association between chronic inflammation and cancer is found in the heightened predisposition for cancer of patients suffering from ulcerative colitis (UC) and Crohn’s disease of the colon, the major forms of idiopathic inflammatory bowel disease (McLarnon, 2011; Risques et al., 2011). Extensive and chronic UC leads to a 19-fold increase in risk for colon cancer, and about 5% of UC patients develop tumors (Gillen et al., 1994). And pancreatic inflammation is a key risk factor for pancreatic cancer (Maisonneuve and Lowenfels, 2002; Raimondi et al., 2010). Another major disease linked to chronic inflammation is gastric cancer, the second leading cause of cancer death worldwide (Qadri et al., 2014; Senol et al., 2014), in which the predisposing inflammation is most often caused by colonization of the gastric epithelium by *Helicobacter pylori*, and chronically infected individuals have an increased risk of developing gastric cancer (Helicobacter and Cancer C, 2001; Meira et al., 2008).

During inflammation, there are increased levels of reactive oxygen and nitrogen species (RONS), which can induce cytotoxic and mutagenic DNA lesions, including abasic sites, oxidized bases (e.g., 8oxoG), deaminated bases (e.g., uracil and hypoxanthine), and ethenoadenine (εA) (Lonkar and Dedon, 2011). In addition to base damage, RONS could also induce DNA double-strand breaks (DSBs). DSBs are among the most toxic of DNA lesions. Moreover, DSBs can also be potently mutagenic due to the potential loss of vast stretches of chromosomes if not accurately repaired (Hoeijmakers, 2009; Maynard et al., 2009). As an unwanted result, these wide range of genomic alterations, including point mutations, copy number changes, and rearrangements, can lead to the development of cancer (Meyerson et al., 2010).

Genomic sequencing has developed to be an effective alternative to locus-specific and gene-panel tests in a research setting for establishing a new genetic basis of disease (de Ligt et al., 2012; Yang et al., 2013). Whole-exome sequencing (WES) is a next-generation technology to determine the variations of the coding regions (exons) of a genome. WES provides coverage of more than 95% exons, which contains 85% disease-causing mutations in many disease-predisposing single-nucleotide polymorphisms (SNPs) throughout the genome (Kaname et al., 2014; Rabbani et al., 2014). Somatic mutations in tumor genome are extensively explored, while the characterization of chronic inflammation-induced somatic mutation via WES approaches is not deeply explored yet, especially the quantitative expansion of different types of genomic alteration during a certain period of inflammation.

Epigenetic alterations, in particular alterations in DNA methylation, are involved in inflammation-associated carcinogenesis (Hartnett and Egan, 2012). Studies have found hypermethylation for p14^ARF^, p16^INK4a^, estrogen receptor, and many other genes in human patients with colitis-associated cancers (Tominaga et al., 2005; Dhir et al., 2008; Wang et al., 2008; Yu et al., 2014). However, how DNA methylation alterations contribute to inflammation-associated carcinogenesis is still unclear. Reports showed that methylation in the promoter region of the upstream area could inhibit gene expression, while gene body methylation was positively correlated with gene expression, which prevented transcription from being too active and related to gene disorder (Ehrlich, 2009; Ndlovu et al., 2011). Therefore, it is valuable to understand the mechanism of upstream cell signal and gene body methylation crosstalk. It can be a guide to understand the pathological and regulation mechanism in the process of inflammation to carcinogenesis.

Here, we present the characteristic of the somatic variation of exome in colon mucosa cells of three chronic colitis mice via WES approaches, the colitis was induced via dextran sulfate sodium (DSS) repeatedly for 40 weeks, and age-matched and no-DSS-treated mice serve as the control mice. In addition, the exome of 8-week-old mouse colon mucosa cells was also sequenced, which is compared with the exome of the 56-week-old (56W) mice to evaluate accumulation of somatic mutation through aging. Our data showed that there was no significant difference in quantification of SNP/insertion or deletion (InDel) mutation between colitis and the control mice, and the similar result appeared in the copy number variation (CNV) number. Furthermore, we found that the SNP/InDel number was obviously elevated in the older mice compared with the young mice, suggesting that aging could make significant contribution to accumulation of somatic mutation. We also performed the DNA methylation sequencing between control and DSS group in 56W mice to explore how DNA methylation alterations in chronic inflammation act upon carcinogenesis. Differentially methylated region (DMR) assay indicated methylation in the upstream, downstream, and gene body regions was significant different. Subsequently, functional analysis showed that differentially methylated genes in chronic inflammation are enriched in the signal pathways related to carcinogenesis. These all suggest that a part of genes related to cancer development appears to have both genetic and epigenetic alterations by chronic inflammation.

## Methods

### Dextran Sulfate Sodium-Induced Chronic Colitis

Distilled water with 2.5% DSS replaced the distilled water for 7 days, during which colitis was induced and the mouse body weight decreased by about 18–20%. Then at the 8 day, DSS water was replaced by distilled water for another 7 days, and then the distilled water was replaced by 2.5% DSS water again. This kind of procedure (7 days of water with 2.5% DSS/7 days of distilled water) was repeatedly performed for 20 times, and the mouse body weight is monitored weekly. Eight weeks after the last DSS treatment procedure, mice were sacrificed for colon mucosa cells and muscularis mucosae isolation.

### DNA Quantification and Qualification

DNA degradation and contamination were monitored on 1% agarose gels. Then DNA purity was checked using the NanoPhotometer spectrophotometer (IMPLEN, Westlake Village, CA, United States). Subsequently, DNA concentration was measured using Qubit DNA Assay Kit in Qubit 2.0 Flurometer (Life Technologies, Carlsbad, CA, United States). Fragment distribution of DNA library was measured using the DNA Nano 6000 Assay Kit of Agilent Bioanalyzer 2,100 system (Agilent Technologies, Santa Clara, CA, United States).

### Whole-Exome Sequencing Library Generation

A total amount of 1 μg of genomic DNA per sample was used as input material for the DNA sample preparation. Sequencing libraries were generated using Agilent SureSelect Mouse All Exon Kit (Agilent Technologies, Santa Clara, CA, United States) following the manufacturer’s recommendations, and index codes were added to attribute sequences to each sample. Briefly, fragmentation was carried out by hydrodynamic shearing system (Covaris, Woburn, MA, United States) to generate fragments of 180–280 bp. Remaining overhangs were converted into blunt ends via exonuclease/polymerase activities, and enzymes were removed. After adenylation of 3′ ends of DNA fragments, adapter oligonucleotides were ligated. DNA fragments with ligated adapter molecules on both ends were selectively enriched in a PCR. After PCR, library hybridize with liquid phase with a biotin-labeled probe and then use magnetic beads with streptomycin to capture the 220,000 exons within 24,000 genes. Captured libraries were enriched in a PCR to add index tags to prepare for hybridization. Products were purified using AMPure XP system (Beckman Coulter, Beverly, MA, United States) and quantified using the Agilent high-sensitivity DNA assay on the Agilent Bioanalyzer 2,100 system.

### Whole-Exome Sequencing and Quality Control

The original image data generated by the sequencing machine were converted into sequence data via base calling (Illumina pipeline CASAVA v1.8.2) and then subjected to quality control (QC) procedure to remove unusable reads: 1) the reads contain the Illumina library construction adapters; 2) the reads contain more than 10% unknown bases (N bases); and 3) one end of the read contains more than 50% of low-quality bases (sequencing quality value ≤ 5).

### Whole-Exome Sequencing Read Mapping

Sequencing reads were aligned to the reference genome using BWA with default parameters. Subsequent processing, including duplicate removal was performed using samtools and PICARD (http://picard.sourceforge.net).

### Variant Detection and Annotation

The raw SNP/InDel sets are called by samtools with the parameters as “-q 1 -C 50 -m 2 -F 0.002 -d 1,000.” Then we filtered these sets using the following criteria: 1) the mapping quality >20 and 2) the depth of the variate position >4. BreakDancer and CNVnator were used for structural variation (SV) and CNV detections, respectively. ANNOVAR was used for functional annotation of variants. The UCSC known genes were used for gene and region annotations.

### Library Construction and Methylated Sequencing

After extraction of genomic DNAs of samples, first, the sample is tested for quality. After the sample quality is qualified, the DNA libraries for bisulfite sequencing were carried out. Specific steps are as follows: the genomic DNA first ultrasound interrupted into the 100–300 bp by Sonication (Covaris) and purified with MiniElute PCR Purification Kit (QIAGEN, Valencia, CA, United States). DNA fragment terminal repaired, 3′ end plus “A” nucleotide base connect the sequencing connector. Methylated sequencing adapters ligate to the genomic fragments. Using ZYMO EZ DNA Methylation-Gold kit ligates methylated sequencing adapters. After desalting treatment, the adhesive is recycled, and the library fragment size selection was performed. Select library fragment size again after PCR amplification. After the construction of the library was completed, the quality inspection of the library was performed. The qualified library will be used for sequencing, which uses Illumina HiSeq™ 2,500 by Gene Denovo Biotechnology Co (Guangzhou, China). The original reads were filtered based on the following rules for getting high-quality clean reads: 1) if reads contain more than 10% unknown nucleotides (N), they will be removed; 2) if reads contain more than 40% of low-quality (Q-value ≤20) bases, low-quality reads will be removed.

### Methylation Level Analysis

The standard clean reading map obtained by BSMAP software (version 2.90) was mapped to the mouse reference genome. Self-defined Perl script to call methylated cytosine and the methylation level was calculated based on the percentage of methylated cytosine in the whole genome, in each chromosome (CG) and in different regions of the genome in each sequence context (CHG and CHH). Subsequently, a 2-kb region methylation profile was drawn based on the average methylation level of each 100-bp interval in order to evaluate different methylation patterns in different genomic regions.

### Differentially Methylated Region Analysis

DMRs for each sequence context (CG, CHG, and CHH) between two samples were identified according to the following stringent criteria: 1) more than five methylated cytosines in at least one sample; 2) more than 10 read’s coverage for each cytosine, and more than four reads for each methylated cytosine; 3) region length is between 40 bp and 10 kb; 4) the distance between adjacent methylated sites <200 bp; 5) the fold change of the average methylation level >2; and 6) Pearson’s chi-square test (χ^2^) value *p* ≤ 0.05. The putative DMRs overlapping at adjacent 2 kb (upstream or downstream) or body regions of genes or transposable elements (TEs) were sorted out for further study.

### Enrichment Analysis of Functional Differently Methylated Region-Related Genes

Significant enrichment analysis was based on Kyoto Encyclopedia of Genes and Genomes (KEGG) pathway (http://www.kegg.jp/), and hypergeometric test was applied to find the pathway of significant enrichment in the DMR-related genes compared with the whole genome background. After multiple examination and correction, the pathway of Q-value 0.05 was defined as the pathway of significant enrichment in the differential expression gene. Pathway (*p*-value ≤0.05, q-value <0.05) was used to analyze whether specific DMRs affect gene’s enrichment.

## Result

### Establishing Chronic Inflammation Model and Strategies for Genomic Sequencing Assay

**FIGURE 1:**
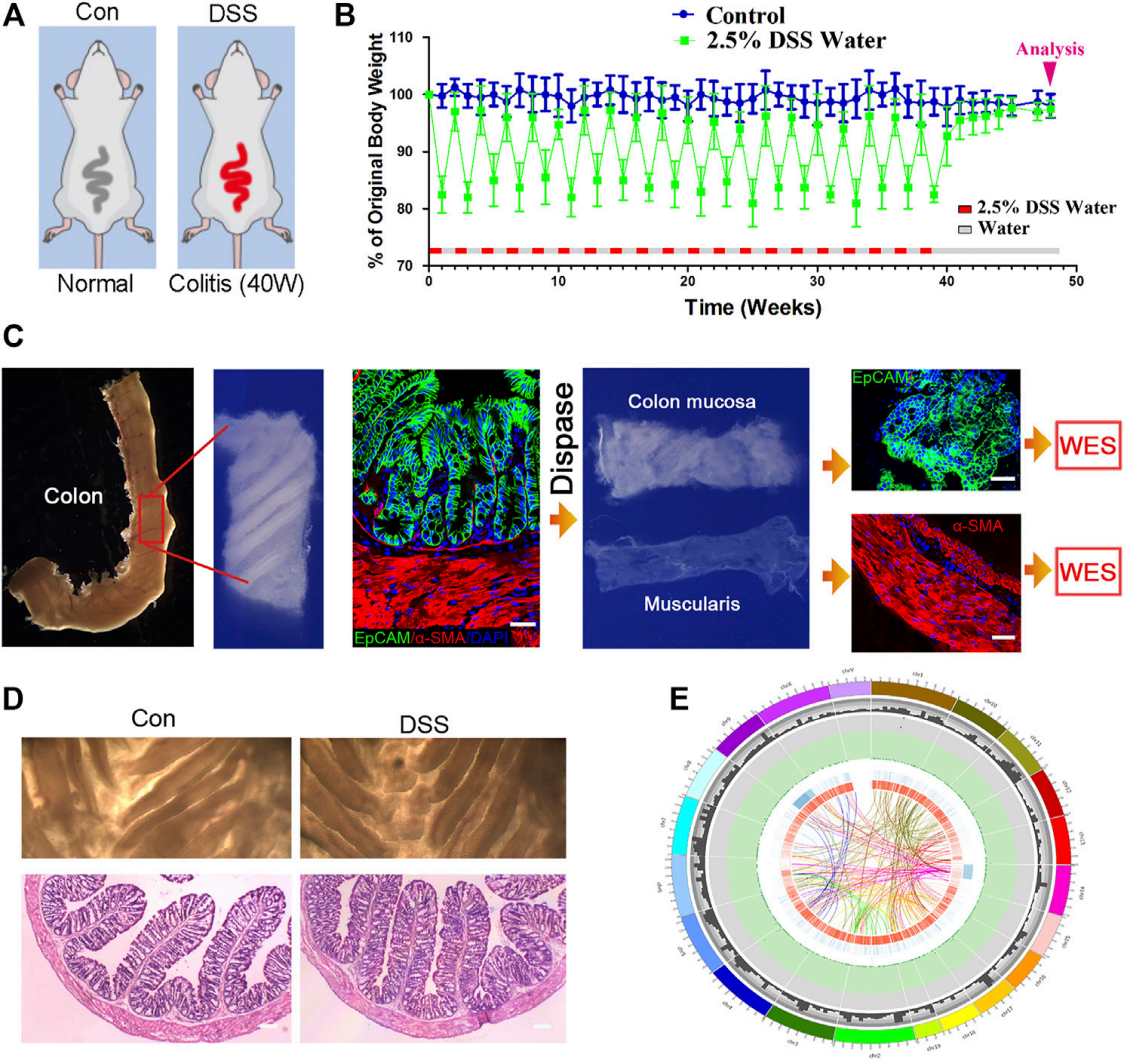
Establishing chronic inflammation model for genomic sequencing assay. **(A, B)** Schematic for dextran sulfate sodium (DSS)-induced colitis, and the procedure of DSS treatment to induce chronic colitis. *n* = 12 for DSS-treated mice and *n* = 10 for control mice. **(C)** The procedure of colon mucosa and muscularis isolation, and the isolated mucosa and muscularis were subjected to whole-exome sequencing (WES). Scale bar: 50 μm. **(D)** Colons were separated in both DSS-treated mice and the age-matched control mice; no obvious morphological difference was found the two groups with dissecting microscope, neither in histopathology. *n* = 6 for both control and DSS-treated mice, and representative results are shown. Scale bar: 50 μm. **(E)** Circos imaging of the overview result of WES, showing (from outside to inside) the length of the genome, the number of genes within 10 M, the frequency of single-nucleotide polymorphisms (SNPs) within 1M (red squares over 0.0015 and green triangles below 0.0005), INS (insertion), INV (inversion), ITX (intrachromosomal translocation), and CTX (interchromosomal translocation).

DSS-induced colitis is a wildly used model for the colitis study, in which the chronic DSS colitis could last over 2 months (Wirtz et al., 2007). While a much longer period of chronic colitis that could last over 10 months was desired in this study, to achieve this kind of chronic colitis, we adjusted DSS administration procedure, as follows: distilled water with 2.5% DSS replaced the distilled water for 7 days, during which colitis was induced and the mouse body weight decreased about 20%; and at the eighth day, DSS water was replaced by distilled water for another 7 days, the mouse body weight was restored, and the distilled water was replaced by 2.5% DSS water again. This kind of procedure (7 days of water with 2.5% DSS/7 days of distilled water) was repeatedly performed for 20 times, the mouse body weight was monitored weekly, and 48 weeks later, the mice were sacrificed for colon mucosa and muscularis mucosae isolation (Figures 1A,B). Colons were separated in both DSS-treated mice and the age-matched control mice. To separate the colon mucosa tissue from the muscularis mucosae tissue, we digested the colon tissue in 0.4% Dispase II at 37°C for 1.5 h and separated the mucosa and muscularis mucosae layer via dissecting forceps. Then tissue section and immunofluorescence staining were performed on the isolated mucosa and muscularis mucosae layer to validate that the mucosa and muscularis mucosae layer are isolated completely and clearly (Figure 1C). No obvious morphological difference was found between the two groups assayed by dissecting microscope, neither histopathologically with H and E assay (Figure 1D). And then the tissues were subjected to DNA isolation and WES. Approximately 20G clean data were generated for each sample, and the average depth is over 200 (>200X). And then the generated reads were compared with reference genome (mm9 mouse genome, related to Agilent mouse exon kit), to evaluate the genomic variations in each individual. The genomic variations of individuals include SNPs, small InDels, and larger-scale variations; and CNV, which generally refers to large-scale (>1 kb) chromosomal copy number changes, e.g., amplifications or deletions, were compared with a reference genome. A overall analysis graph is presented in Figure 1E, which includes the SNP frequency in 1 M, and also the INS (insertion), INV (inversion), ITX (intrachromosomal translocation), and CTX (interchromosomal translocation).

### Quantitative Assay of Single-Nucleotide Polymorphism/Insertion or Deletion in the Colitis Tissue

**FIGURE 2:**
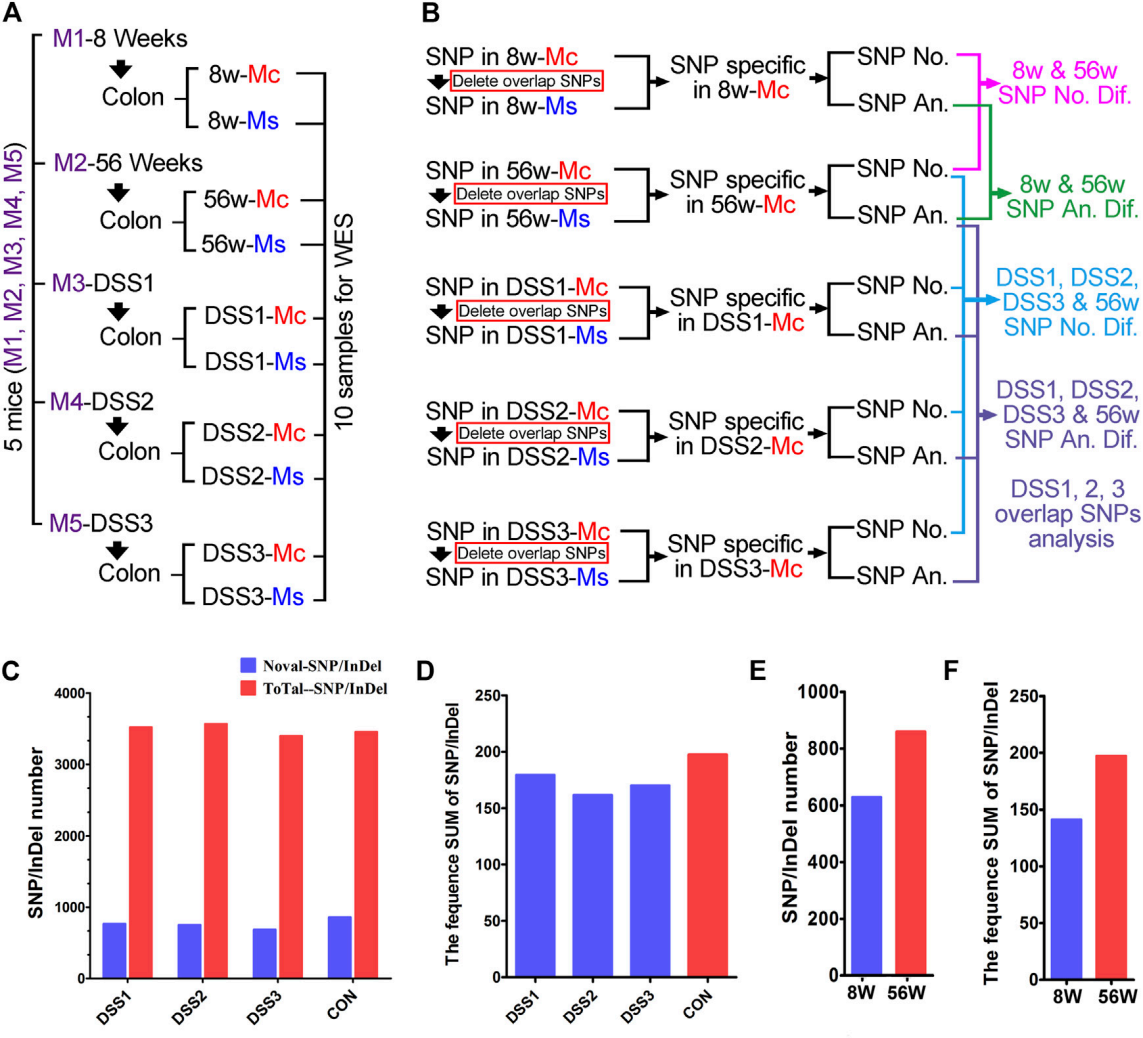
Comparative and quantitative assays of single-nucleotide polymorphism/insertion or deletion (SNP/InDel) in the colitis tissue. **(A)** Ten samples from five mice were used for whole-exome sequencing (WES), mucosa (Mc) and muscularis (Ms) isolation and sequenced individually. **(B)** Comparative assay strategy for the WES result; SNP/InDel from the Mc and Ms of same mice was compared to wipe off the germline mutation. Both statistical and annotative assays were performed on the SNP/InDel specific in Mc from different mice. **(C)** The total SNP/InDel number in the mucosa of three dextran sulfate sodium (DSS)-induced colitis mice is 3,521/3,570/3,400; for the no colitis mouse, the SNP/InDel number is 3,456. The novel somatic SNP/InDel number in the three DSS-induced colitis mice is 769/753/687; in age-matched (56W) no-colitis control mouse, the SNP/InDel number is 860. **(D)** SNP/InDel frequency SUM is 179/162/170 in three DSS-induced colitis mice and is 195 in the control mice. **(E)** The SNP/InDel number is obviously elevated in the older mouse (56 weeks, SNP/InDel: 860) compared with the young mouse (8 weeks, SNP/InDel: 629). **(F)** The SNP/InDel frequency SUM in 56W mouse was obviously increased compared with that in the 8W mouse.

Ten samples from five mice were prepared for WES in this study; the five mice (close breeding and same generation) included an 8-week-old mouse (young mouse), a 56W mouse (old mouse, also served as the age-matched mouse for colitis mice), and three colitis mice (56W). The mucosa (Mc) and muscularis mucosae (Ms) tissue were separated from the colon of the abovementioned five mice (Figure 2A). And concerning the germline mutation contained in the total SNP/InDel, the exome of muscularis mucosae was also sequenced to exclude the germline mutation and then to highlight the somatic mutation generated in the mucosa (the mutations existing in both the mucosa and muscularis were taken as the germline mutation). The SNP/InDel in the mucosa excluded the SNP/InDel muscularis of the same mice and was defined as the SNP/InDel specific in the mucosa (Figure 2B). And then the number and annotation of SNP/InDel specific in different mucosae were comparatively assessed (Figure 2B). The total SNP/InDel number (compared with the reference sequence) in the mucosa of three colitis mice is 3,521, 3,570, and 3,400, respectively, while in the age-matched no-colitis mouse, the SNP/InDel number is 3,456, indicating that there was no significant quantitative difference in total SNP/InDel mutation between the colitis and control tissues (Figure 2C). The somatic (novel) SNP/InDel number in the three DSS-induced colitis mice is 769/753/687, while in the age-matched (56W) control mouse, the SNP/InDel number is 860 (Figure 2C). To show an SNP/InDel profile that could take the SNP/InDel frequency into account, we calculated the summation (SUM) of all the somatic SNP/InDel frequencies in each individual, and we compared the SNP/InDel frequency SUM between different individuals. Similar to the SNP/InDel number, the SNP/InDel frequency SUM in three DSS-induced colitis mice was not obviously different from that of the control mouse, as SNP/InDel frequency SUM is 179/162/170 in three DSS-induced colitis mice and 195 in the control mice (Figure 2D). These data suggest that chronic inflammation could not significantly increase the accumulation of SNP/InDel number as well as its frequency. Similarly, the somatic CNV number in the mucosa of the colitis mice was also not significantly different from that of the control mice (Figure 2E).

Even though somatic SNP/InDel/CNV number was not increased by chronic inflammation, we found that the SNP/InDel number was obviously elevated in the older mouse (56W, SNP/InDel: 860) compared with the young mouse (8 weeks old, SNP/InDel: 629) (Figure 2F). Thus, this result suggests that aging makes significant contribution to the accumulation of somatic mutation in the individuals.

### Annotation of Single-Nucleotide Polymorphism/Insertion or Deletion Accumulated in the Old and Dextran Sulfate Sodium-Treated Mice

**FIGURE 3:**
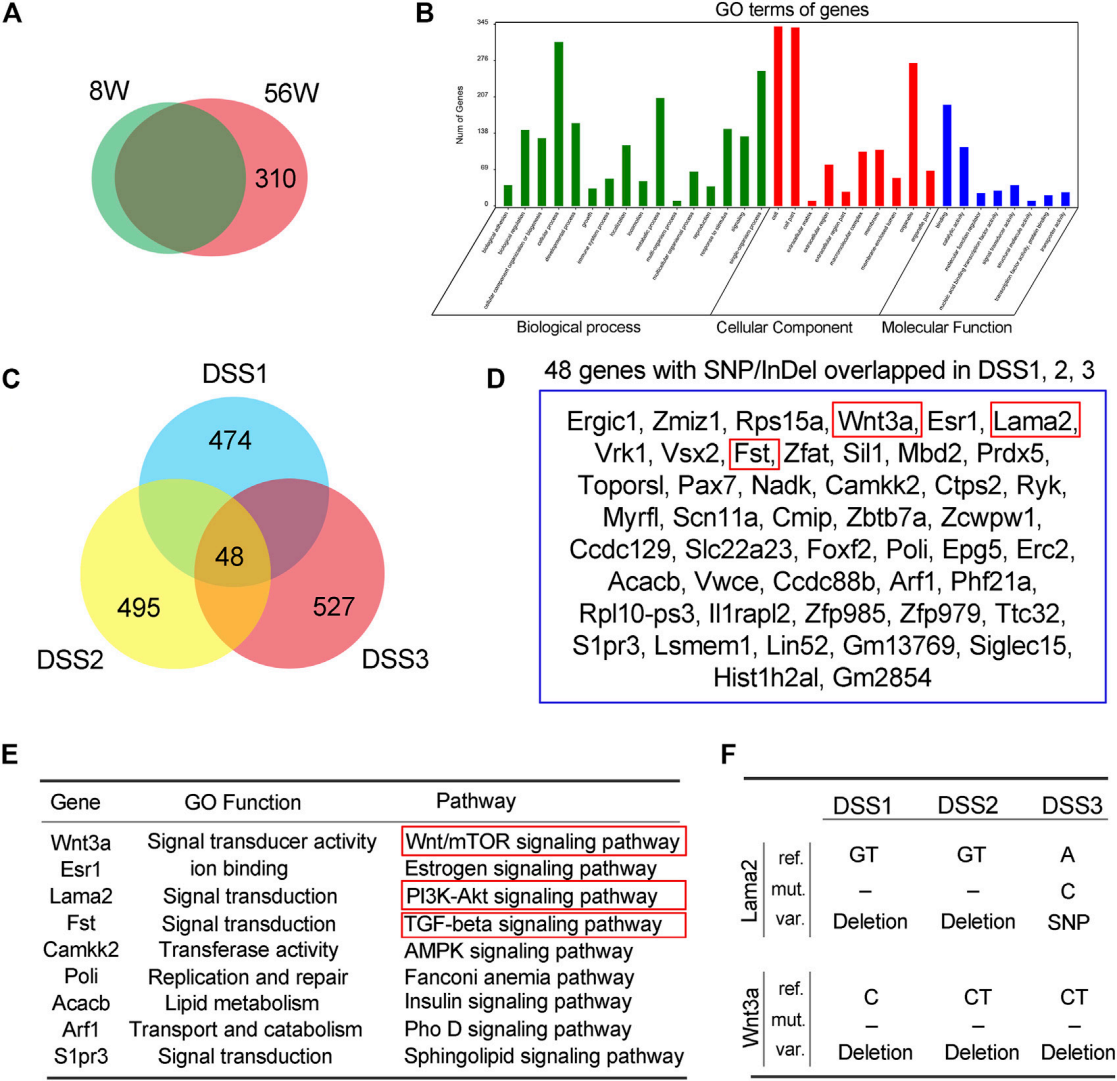
Annotation of single-nucleotide polymorphism/insertion or deletion (SNP/InDel) accumulated in the old and dextran sulfate sodium (DSS)-treated mice. **(A)** There were 310 SNP/InDel unique in 56W mice mucosa. **(B)** Gene Ontology (GO) enrichment showed that these genes were classified into the cellular function of biological process, cellular component, and molecular function. **(C)** There were 769/753/687 somatic (novel) SNP/InDel in the three colitis mice, and this SNP/InDel was annotated into 474/495/527 genes; among these genes from different mice, 48 genes were overlapped. **(D, E)** The 48 genes that were overlapped in three colitis mice. Among these 48 genes, Wnt3a, Lama2, and Fst exert signal transduction function in the Wnt/mTOR, PI3K–AKT, and TGF-beta signaling pathways, respectively. **(F)** Lama2 mutation among the three DSS-treated mice mucosa, two of them were GT deletion mutation, and the other was SNP, while all the Wnt3a mutations in three DSS-treated mucosa were deletion mutation.

To get a further insight on the functional profile of SNP/InDel that uniquely accumulated in old (56W) or DSS-treated mouse colon mucosa, further assay was performed. Comparative assay of the SNP/InDel showed that there were 310 SNP/InDel unique in 56W mouse mucosa; Gene Ontology (GO) enrichment showed that these genes were classified into the cellular function of biological process, cellular component, and molecular function (Figures 3A,B). There were 769/753/687 somatic (novel) SNP/InDel in the three colitis mice, and this SNP/InDel was annotated into 474/495/527 genes; among these genes from different mice, 48 genes were overlapped (Figure 3C), listed in Figure 3D. Various signaling pathways regulate cellular proliferation, differentiation, and immortalization of colorectal cancer, especially Wnt/β-catenin, PI3K/AKT/mTOR and TGF-beta/Smad signaling (Pandurangan et al., 2018). Among these 48 genes, Wnt3a, Lama2, and Fst exert signal transduction function in the Wnt/mTOR, PI3K–AKT, and TGF-beta signaling pathways, respectively (Figure 3E). Lama2 is a tumor suppressor by changes in its expression and methylation patterns and can modulate PTEN to exert effects on PI3K/AKT signaling (Wang et al., 2019). For Lama2 mutation among the three DSS-treated mouse mucosae, two of them were GT deletion mutation, and the other was SNP (A–C). Wnt3a is a Wnt protein that activates the canonical Wnt pathway and promotes colon cancer progression (Clevers, 2006; Qi et al., 2014), while all the Wnt3a mutations in three DSS-treated mucosae were deletion mutation (one is C deletion and other two is CT deletion) (Figure 3F).

### The Impact of Chronic Inflammation Upon the DNA Methylation Profile in Colon Mucosa

**FIGURE 4:**
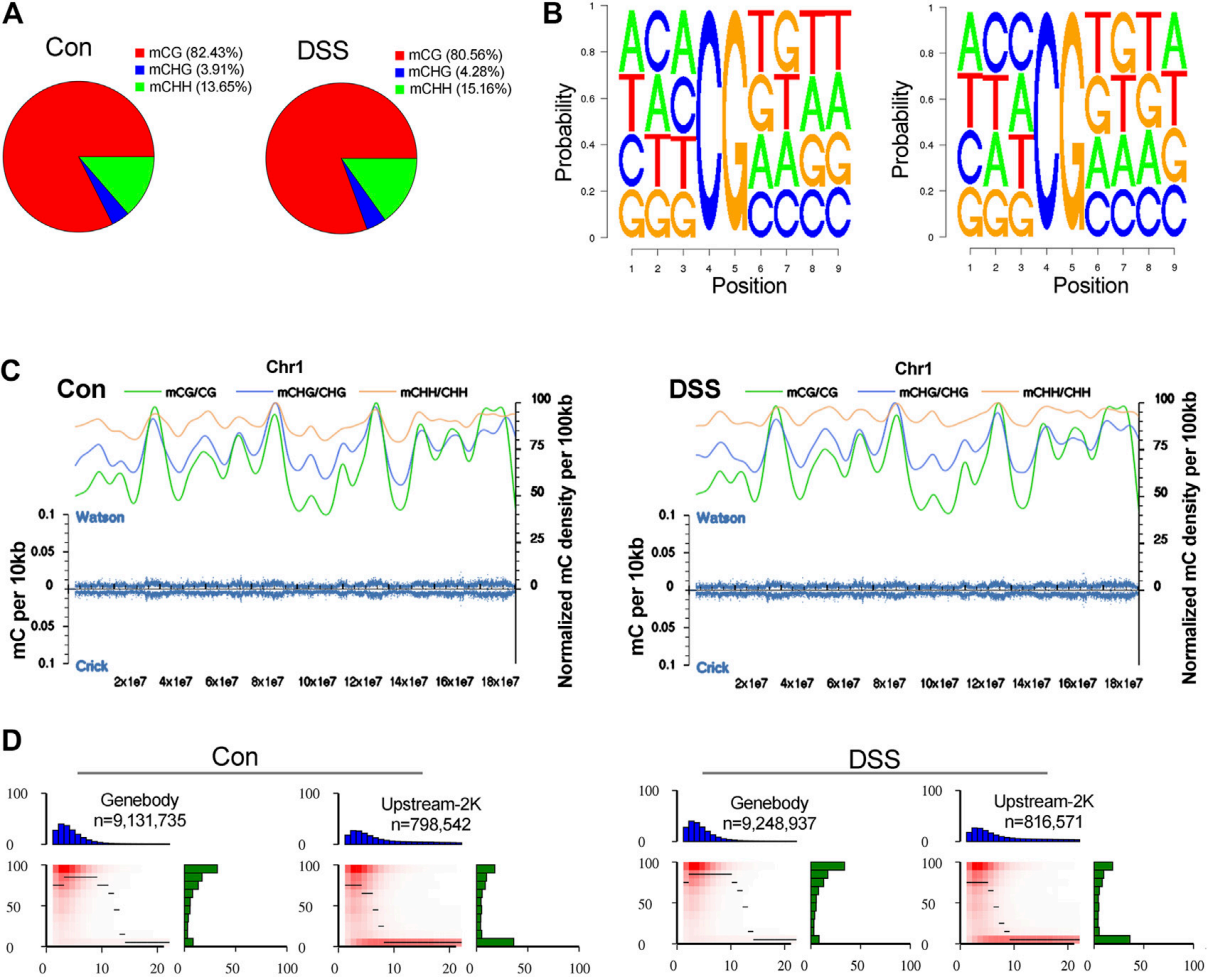
The impact of chronic inflammation upon the DNA methylation profile in colon mucosa. **(A)** The ratio of different types of mC (mCG, mCHG, and mCHH) in dextran sulfate sodium (DSS)-treated and control mice were assessed, in which mCG was 82.43 and 80.56% in control and DSS-treated mice, respectively. **(B)** The sequence characterization of the nucleotides near methylation C of CG, by counting the 9-bp base near CG bases and comparing the base of CG and mCG in the whole genome. **(C)** mC density in control and DSS-treated mice chromosome 1. **(D)** The characteristics of methylation patterns in different gene regions are represented by heat map.

**FIGURE 5:**
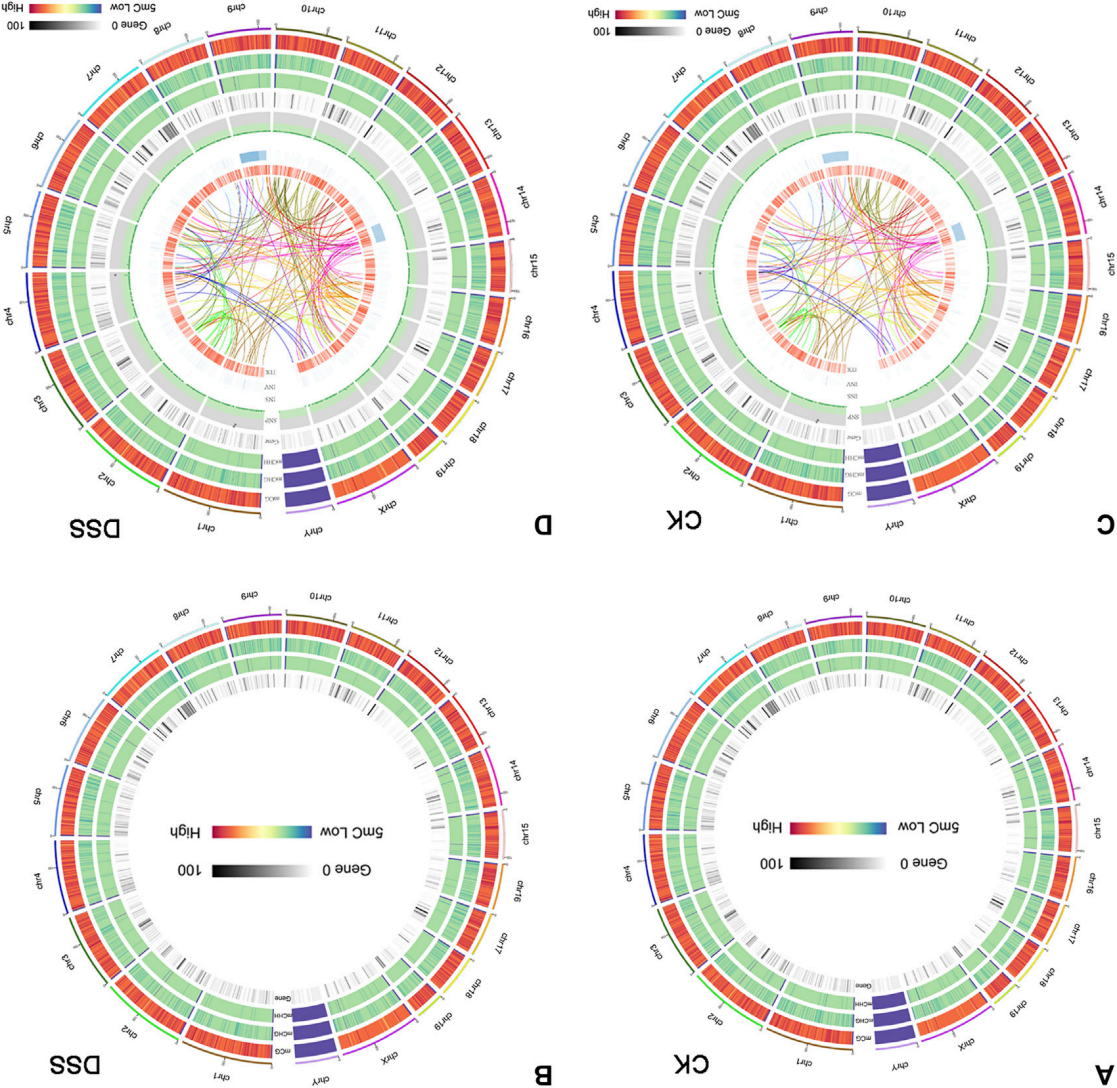
The overview of DNA methylation in control and dextran sulfate sodium (DSS)-treated mice and the conjoint analysis of DNA methylation and gene mutation. **(A, B)** The genomic distribution of mCG, mCHG, and mCHH in two different groups of mice was presented in a Circos graphs. **(C, D)** The conjoint analysis of the DNA methylation and whole-exome sequencing (WES) result.

To explore the epigenetic profile in the colon mucosa with chronic inflammation, bisulfite sequencing was performed. In the genomic DNA, C bases can be classified into three groups according to their sequence features as CG, CHG, and CHH. In methylated C, the proportions of these three sequence types vary among species. Thus, the number of each type of mC (mCG, mCHG, and mCHH), and their share of total mC sites, could reflect the characteristics of the genome-wide methylation profile. The ratio of different types of mC in DSS-treated and control mice was assessed, in which mCG was 82.43 and 80.56% in control and DSS-treated mice, respectively (Figure 4A). The sequence features of the bases near the methylation sites within the whole genome are instructive to reflect the sequence bias of methylation. Counting the 9-bp base near CG bases and comparing the base of CG and mCG in the whole genome enabled to obtain the sequence bias characteristic of methylation. The sequence characterization of the nucleotides near methylation C of CG is presented in Figure 4B. In addition, by analyzing the density distribution of mC at the chromosome level, we obtained the centralization bias of the methylation at the macro level. The mC density in each chromosome was assessed individually, and the result of mC density in control and DSS-treated mouse chromosome 1 is presented (Figure 4C). Different genomic regions have different biological functions; to get further insight on the mC distribution feature, the distribution of methylation status is represented by heat map, which visualized the characteristics of methylation patterns in different gene regions (Figure 4D). The genomic distribution of mCG, mCHG, and mCHH in two different groups of mice is presented in circle graphs (Figures 5A,B). In addition, the conjoint analysis of the DNA methylation and WES result was performed; the profile of both DNA methylation and gene mutation in control and DSS-treated mice is presented (Figures 5C,D).

### Annotation of the Differently Methylated Regions in Chronic Inflammation Mouse Genomic

**FIGURE 6:**
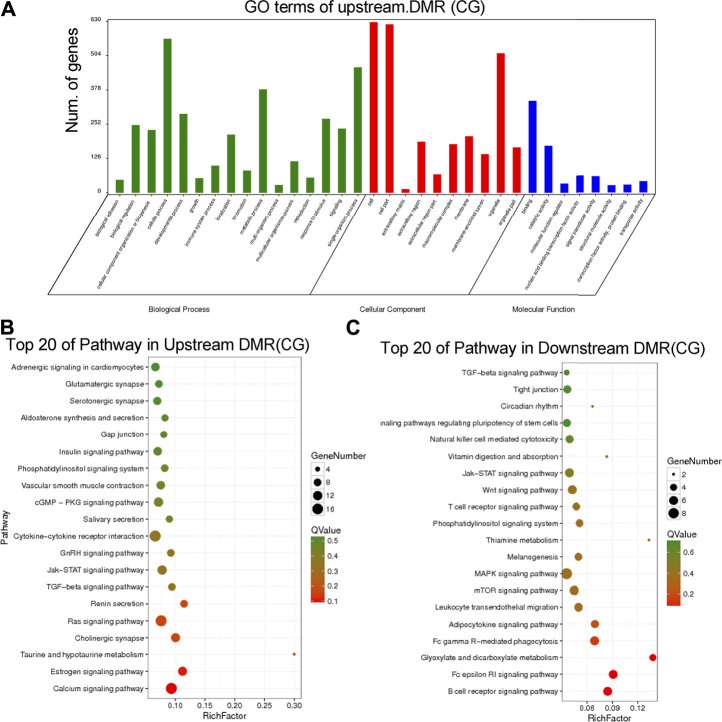
Annotation of the differently methylated regions (DMRs) in chronic inflammation mice genomic. **(A)** Gene Ontology (GO) enrichment result showed the DMRs enriched in biological process, cellular component, and molecular function. **(B)** The top 20 Kyoto Encyclopedia of Genes and Genomes (KEGG) pathway of upstream DMR (CG)-related genes. **(C)** The top 20 KEGG pathway of downstream DMR (CG)-related genes.

**FIGURE 7:**
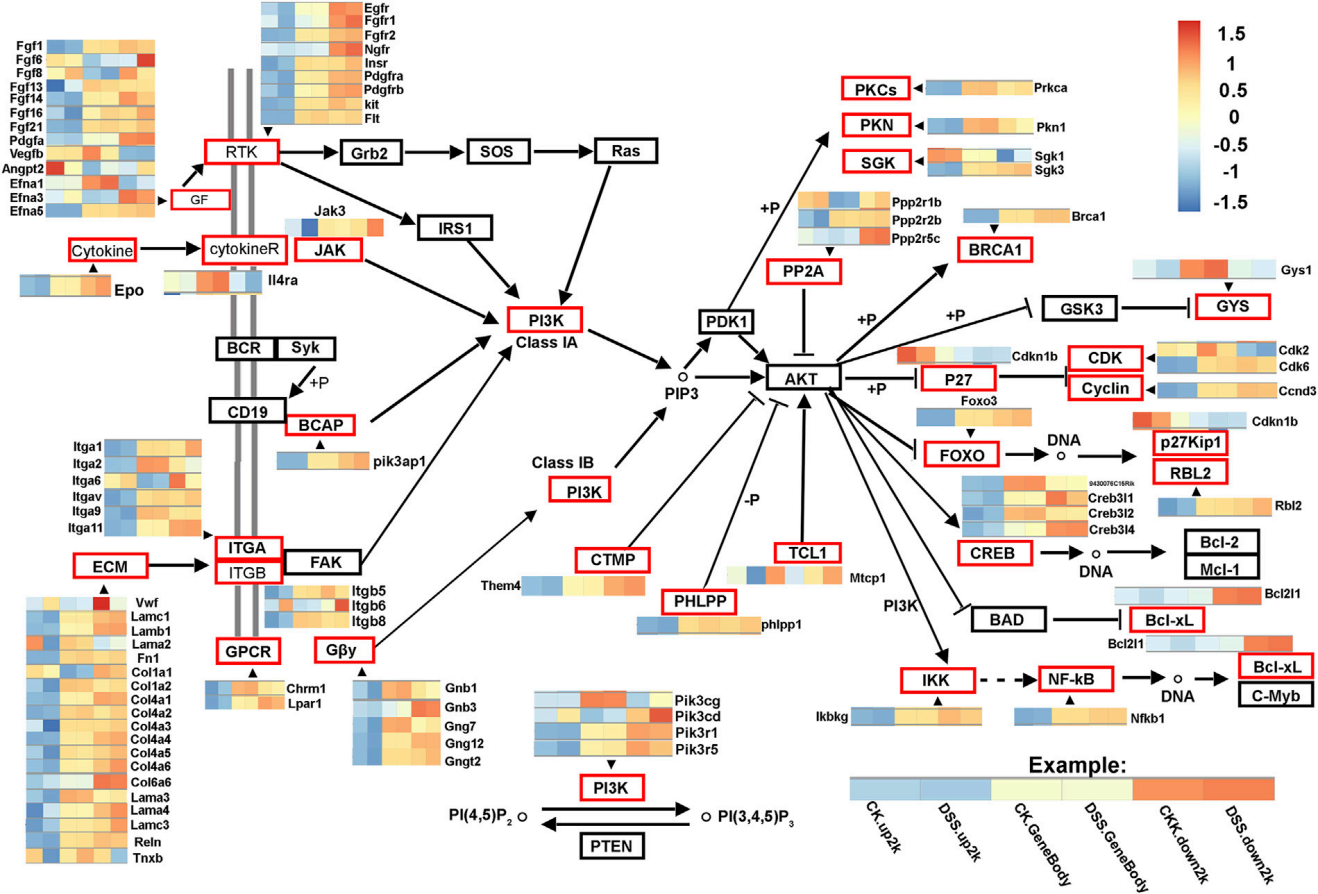
DNA methylation in different genes of PI3K/AKT signaling pathways. The network shows differentially DNA methylated genes of PI3K/AKT signaling pathways. The gene regions (CK.gene body and DSS. gene body), 2 kb upstream of the gene regions (CK.up2k and DSS. up2k), and downstream of the gene regions (CK.down2k and DSS. down2k) in control and dextran sulfate sodium (DSS)-treated mice are analyzed. The differentially DNA methylated genes are marked in red boxes.

To further interpret the DNA methylation result, analysis of the DMR was performed. The DMRs that meet a certain condition in the same position of both control and DSS-treated mice were searched, and the difference in methylation level in this region is greater than 2 as DMRs in this study. Finally, according to the DMR and gene region (including 2 kb upstream of the gene and 2 kb downstream of the gene) on each chromosome, the genes related to differential methylation were determined, and GO and pathway enrichment analysis of these genes were performed. GO enrichment result showed the genes that most significantly enriched in biological process including cellular process, metabolic process, and single-organism process. And the genes in cell, cell part, and organelle were significantly enriched in cellular component. Genes of binding and catalytic activity were significantly enriched in molecular function (Figure 6A). Different genes interact with each other to executive certain biological function, pathway assay helps understand gene function, and KEGG is the major public database for pathway assay. KEGG pathway enrichment could determine biochemical and metabolic participation of DMR-related genes. In this study, the KEGG assay showed that upstream DMR (CG)-related genes are enriched to pathways including Jak-STAT signaling pathway, TGF-beta signaling pathway, and Ras signaling pathway (Figure 6B). And the downstream DMR (CG)-related genes are enriched to pathways including Wnt signaling pathway, MAPK signaling pathway, and mTOR signaling pathway (Figure 6C). The KEGG enrichment of DMR-related genes in different signaling pathways was assessed independently. DNA methylation in different genes of PI3K/AKT signaling pathways and network of gene regulation is shown in Figure 7. In conclusion, the data of this study suggest that chronic inflammation showed little influence on genetic stability; no significant mutations were accumulated in chronic tissue, while the chronic inflammation did have a certain impact on the DNA methylation of colon mucosa tissue.

## Discussion

The relationship between inflammation and cancers has been studied for over 150 years, and accumulated researches support that chronic inflammatory diseases are related to cancers (Balkwill and Mantovani, 2001; Coussens and Werb, 2002; Philip et al., 2004). As early as 1,863, Virchow indicated that cancers tended to occur at sites of chronic inflammation. Lately, it turned out that acute inflammation contributed to the regression of cancer. However, accumulated epidemiologic studies support that chronic inflammatory diseases are frequently associated with increased risk of cancers. Our understanding of the association between chronic inflammation and cancer is mostly illustrated by inflammatory bowel disease and colon carcinogenesis. Previous report shows that there was an 18-fold increase in the risk of developing colorectal cancer in extensive Crohn’s colitis and a 19-fold increase in risk in extensive UC when compared with the general population, matched for age, sex, and years at risk (Gillen et al., 1994). And increased cancer incidence is associated with increased duration of the inflammation. On basis of the toxic effect of inflammation/RONS on DNA, in this study, we hypothesize that chronic inflammation would result in somatic mutation accumulation, which thereby increases the risk of carcinogenesis. While inconsistent with highly increased cancer development risk in inflammation, there was no somatic mutation increase observed during chronic inflammation, thus increasing the somatic mutation dislike to be the mediator of inflammation-induced cancer developing risk.

Previous reports indicate that the number of ε-base lesions and 8oxoG increased in the colons of mice following a single DSS treatment; in addition, consistent with the ability of Aag (alkyladenine DNA glycosylase) to excise both εA and 8oxoG (Bartsch and Nair, 2002; Meira et al., 2008), inflammation-induced εA, εC, and 8oxoG increased more dramatically in the Aag-deficient mice, since Aag could recognize the DNA damage and initiate base excision repair. Moreover, in the mice with deficiency in three DNA repair proteins (Aag^−/−^/Alkbh2^−/−^/Alkbh3^−/−^ triple-knockout), a single cycle of DSS-induced colitis resulted in absolute lethality of these mice (Calvo et al., 2012). These studies indicate the crucial role of the DNA repair proteins in both tumor suppression and tissue homeostasis. Despite the increased levels of toxic and mutagenic εA and 1, *N* ^*2*^-εG in colon mucosa cells following DSS treatment, our data suggest that these DNA lesions do not ultimately contribute to the accumulation of somatic genomic alteration, including the SNP/InDel and CNV; presumably, there is redundant potential of DNA repair proteins, which is enough to compensate the increased DNA damage/repair activity during the inflammation, thus finally not resulting in the obviously increased DNA mutation. Collectively, increasing the accumulation of somatic gene mutations does not seem to be the major mechanism of chronic inflammation in promoting the neoplasia; and we proposed that the DNA repair mechanism is efficient enough to repair the increased DNA damage induced by inflammation, thus eliminating the risk of DNA mutation accumulation.

The methylation CpG islands in the promoter region of tumor suppressor genes can silence gene expression and lead to tumorigenesis (Baylin and Jones, 2011). In this process, the activation status of several cancer-related pathways are changed, including Ras, Wnt/β-catenin, PI3K/AKT, and MAPK signaling pathways (Jones and Baylin, 2007; Ying and Tao, 2009; Fattahi et al., 2020). In our study, the differentially methylated genes and gene region (including 2 kb upstream of the gene and 2 kb downstream of the gene) were determined, and GO and KEGG enrichment analysis of these genes were performed. We found that upstream DMR (CG) and downstream DMR (CG)-related genes were enriched to different cancer-related pathways, which pointed their different functions on carcinogenesis.

Tumor formation is a complex process involving many genes and procedures. The process of chronic inflammatory models may be a transition between inflammation and cancer. Our research presents how chronic inflammation transitions to a tumor, what happens on the exon, what goes on in the epigenetic methylation. And our results provide a train of thoughts. The process of studying methylation can predict in advance which proteins will be disorganized. We may be able to reverse the transformation cells by correcting DNA methylation abnormalities. In conclusion, to study methylation and exon sites SNPs in the human disease model is significant for the diagnosis and treatment of chronic inflammation and tumors.

## Data Availability

The whole-exome sequencing data presented in this study have been deposited in the European Variation Archive repository (https://www.ebi.ac.uk/eva), accession number: PRJEB48005; The methylated sequencing data in this paper have been deposited in the OMIX, China National Center for Bioinformation/Beijing Institute of Genomics, Chinese Academy of Sciences (https://ngdc.cncb.ac.cn/omix), accession number: OMIX694.
